# Editorial: TGF-β as a Key Regulator of NK and ILCs Development and Functions

**DOI:** 10.3389/fimmu.2020.631712

**Published:** 2021-01-19

**Authors:** Cristina Bottino, Thierry Walzer, Angela Santoni, Roberta Castriconi

**Affiliations:** ^1^ Laboratory of Clinical and Experimental Immunology, Istituto di Ricovero e Cura a Carattere Scientifico (IRCCS), Istituto Giannina Gaslini, Genoa, Italy; ^2^ Department of Experimental Medicine, University of Genoa, Genoa, Italy; ^3^ Centre International de Recherche en Infectiologie, INSERM U1111 - CNRS UMR5308, Université de Lyon, ENS de Lyon, Université Lyon, Lyon, France; ^4^ Department of Molecular Medicine, Laboratory Affiliated to Istituto Pasteur Italia, Sapienza University of Rome, Rome, Italy

**Keywords:** NK cells, ILC, NKG2D activating receptor, epigenetic mechanisms, chemokine receptors, NK cell metabolism, transforming growth factor-β

TGF-β is a crucial actor in the scenario of immune regulation. Over the years it emerged as a key tolerogenic cytokine capable of preserving normal tissue homeostasis and avoiding tissue damage upon immune responses. TGF-β1 gene knock-out embryos showed a 50% intrauterine death rate, and, if born, died by 3 to 6 weeks because of a fatal multi-organ inflammation (1). Along this line, several studies addressed the role of TGF-β in the regulation of postnatal inflammatory responses, exploring its effect on different cell types of the immune system like Natural Killer (NK) cells, which are characterized by strong anti-tumor cytolytic activity as the result of the cooperation of different activating receptors. These include NKp30 and NKG2D, whose surface expression is severely impaired by TGF-β with a significant impact on immune-surveillance (2).

The present Research Topic hosts an overview by Lazarova and Steinle on the TGF-β-mediated impairment of the NKG2D axis, with implications in anti-tumor immunity and cancer therapies. As discussed by the authors, the impairment of the NKG2D axis represents a biomarker of TGF-β-compromised cytotoxic lymphocytes in cancer patients. TGF-β would act on two sides, decreasing the expression of NKG2D, on immune effectors, and NKG2D ligands on tumor cells (3, 4). Interestingly, TGF-β also acts as a membrane-bound form expressed by T regulatory cells and Myeloid-Derived Suppressor Cells (MDSC) in the tumor microenvironment. Moreover, in mesothelioma patients, TGF-β carrying exosomes inducing down-regulation of NKG2D in NK cells have been recently described (5).

Several observations derived from *in vitro* studies indicate that the rescue of the NKG2D-NKG2DL axis could represent a promising strategy to potentiate anti-tumor immune surveillance in cancer patients (3). In this regard, anti-cancer therapies targeting the TGF-β pathway and different clinical trials are discussed in Lazarova and Steinle’s article. Therapeutic strategies include the use of galunisertib, targeting the TGF-β receptor I (TGF-βRI) kinase activity, which had significant therapeutic effects in phase II clinical trials in patients with pancreatic cancer, hepatocellular carcinoma, and neuroblastoma, in the absence of adverse side effects including cardiac toxicity (6). Other promising clinical approaches, with interesting results in preclinical models, are based on the use of either immune cells transduced with a chimeric antigen receptor (CAR) specific for TGF-β, which dimerizes and transduces signals upon TGF-β binding, or a soluble form of TGF-βRII conjugated with antibodies blocking immune checkpoints. The latter approach is associated with increased tumor infiltration by NK and T lymphocytes (7) in line with the capability of TGF-β of negatively modulating the chemokine receptor repertoire of human NK cells (2). In this context, CX3CR1 has been shown to be one of the chemokine receptors down-regulated by TGF-β in NK cells, an effect involving post-transcriptional regulatory mechanisms such as the induction of miRNA 27a-5p expression (8). This pathway as well as other TGF-β-induced epigenetic modulatory mechanisms are briefly discussed in this topic by Regis et al. The article also discusses the role of other TGF-β-induced miRNAs. These include miR-1245 that causes a variable degree of NKG2D down-regulation depending on individual NKG2D 3’-UTR polymorphisms, and miR146a targeting the transcription factor STAT1 involved in interferons signal transduction. S. Regis also briefly discusses TGF-β-induced changes in the expression of several transcription factors, and the impact of TGF-β-induced epigenetic changes on NK cell metabolism. The latter issue is extensively addressed by Slattery and Gardiner who review the literature concerning the inhibitory role of TGF-β on different metabolic pathways including that regulated by mTOR complexes. The authors discuss the contrasting results describing the regulatory role of TGF-β on mTOR activity, suggesting that discrepancies might depend on the duration of TGF-β conditioning (acute versus chronic) or the type of cytokine milieu (IL-15 versus IL-12/IL-15) used for boosting NK cell metabolism (9, 10). Slattery and Gardiner also briefly discuss the unexpected TGF-β-mediated activation of mTOR in healthy or pathological non-immune cells such as fibroblasts, kidney cells, and cervical carcinoma cells (11). TGF-β conditioning induced an NK cell impairment stronger than Rapamycin, the gold standard compound targeting the mTOR pathway, suggesting possible additional regulatory functions of TGF-β on NK cells metabolism. This hypothesis also arises from a wide literature describing, in cell types different from NK cells, the ability of TGF-β to reduce MYC activity, the Endoplasmic Reticulum -mitochondria Ca2+ signaling and antioxidants production, while increasing reactive oxygen species (ROS). The review by Slattery and Gardiner ends with an interesting perspective on the therapeutic potential of TGF-β blockers to fully recovering the anti-tumor function of NK cells, either present or adoptively transferred in patients.

These therapeutic approaches might also impact, *in vivo*, on the size of the NK cell population since it has been shown that activation of mTOR is essential for the IL-15-driven development of NK cells (12). Moreover, many studies indicate that in peripheral tissues TGF-β might influence the composition of the innate lymphoid compartment since it has been shown to convert NK cells into a less cytotoxic, ILC1-like, population (13–15). In line with these observations, in this Research Topic, Cuff et al. describe the prevalence, in the liver of obese mice, of poorly cytotoxic NK cells with an altered metabolic profile, and a transcriptional profile typical of ILC1. The liver of these mice contains levels of TGF-β higher than those of lean mice, and TGF-β conditioning converts into ILC1-like cells NK cells isolated from lean mice. This suggests that, *in vivo*, TGF-β may be responsible for this peculiar NK cell phenotype and confirms the plasticity of NK cells in response to TGF-β conditioning. Interestingly, the NK cell phenotype described in obese mice partially recapitulates that observed in NK cells from the liver of patients with Non-alcoholic fatty liver disease (NAFLD) and Non-alcoholic steatohepatitis (NASH).

Overall, the article collection in this Research Topic provides a comprehensive overview of several regulatory functions exerted by TGF-β in NK cells, describing well-consolidated effects, and discussing more recent findings highlighting the key regulator role of TGF-β in NK cell development.

## Author Contributions

CB, TW, AS, and RC wrote the editorial. All authors contributed to the article and approved the submitted version.

## Conflict of Interest

The authors declare that the research was conducted in the absence of any commercial or financial relationships that could be construed as a potential conflict of interest.

## References

[1] Shull MM Ormsby I AB K Pawlowski S Diebold RJ Yin M Targeted disruption of the mouse transforming growth factor-beta 1 gene results in multifocal inflammatory disease. Nature (1992) 359(6397):693–9. 10.1038/359693a0 PMC3889166 1436033

[2] Casu B Dondero A Regis S Caliendo F Petretto A Bartolucci M Novel Immunoregulatory Functions of IL-18, an Accomplice of TGF-beta1. Cancers (Basel) (2019) 11(1):75. 10.3390/cancers11010075 PMC6356463 30641867

[3] Ullrich E Koch J Cerwenka A Steinle A New prospects on the NKG2D/NKG2DL system for oncology. Oncoimmunology (2013) 2(10):e26097. 10.4161/onci.26097 24353908 PMC3862635

[4] Zingoni A Vulpis E Loconte L Santoni A NKG2D Ligand Shedding in Response to Stress: Role of ADAM10. Front Immunol (2020) 11:447. 10.3389/fimmu.2020.00447 32269567 PMC7109295

[5] Clayton A Mitchell JP Court J Linnane S Mason MD Tabi Z Human tumor-derived exosomes down-modulate NKG2D expression. J Immunol (2008) 180(11):7249–58. 10.4049/jimmunol.180.11.7249 18490724

[6] Kovacs RJ Maldonado G Azaro A Fernández MS Romero FL Sepulveda-Sánchez JM Cardiac Safety of TGF-beta Receptor I Kinase Inhibitor LY2157299 Monohydrate in Cancer Patients in a First-in-Human Dose Study. Cardiovasc Toxicol (2015) 15(4):309–23. 10.1007/s12012-014-9297-4 PMC4575352 25488804

[7] Lan Y Zhang D Xu C Hance KW Marelli B Qi J Enhanced preclinical antitumor activity of M7824, a bifunctional fusion protein simultaneously targeting PD-L1 and TGF-beta. Sci Transl Med (2018) 10(424):eaan5488. 10.1126/scitranslmed.aan5488 29343622

[8] Regis S Caliendo F Dondero A Casu B Romano F Loiacono F TGF-β1 Downregulates the Expression of CX3CR1 by Inducing miR-27a-5p in Primary Human NK Cells. Front Immunol (2017) 8:868. 10.3389/fimmu.2017.00868 28791023 PMC5524732

[9] Viel S Marquis A Guimaraes FS Loftus R Rabilloud J Grau M TGF-beta inhibits the activation and functions of NK cells by repressing the mTOR pathway. Sci Signal (2016) 9(415):ra19. 10.1126/scisignal.aad1884 26884601

[10] Zaiatz-Bittencourt V Finlay DK Gardiner CM Canonical TGF-β Signaling Pathway Represses Human NK Cell Metabolism. J Immunol (2018) 200(12):3934–41. 10.4049/jimmunol.1701461 29720425

[11] Selvarajah B Azuelos I Platé M Guillotin D Forty EJ Contento G mTORC1 amplifies the ATF4-dependent *de novo* serine-glycine pathway to supply glycine during TGF-β 1-induced collagen biosynthesis. Sci Signal (2019) 12(582):eaav3048. 10.1126/scisignal.aav3048 31113850 PMC6584619

[12] Marçais A Cherfils-Vicini J Viant C Degouve S Viel S Fenis A The metabolic checkpoint kinase mTOR is essential for IL-15 signaling during the development and activation of NK cells. Nat Immunol (2014) 15(8):749–57. 10.1038/ni.2936 PMC4110708 24973821

[13] Gao Y Souza-Fonseca-Guimaraes F Bald T Ng SS Young A Foong Ngiow S Tumor immunoevasion by the conversion of effector NK cells into type 1 innate lymphoid cells. Nat Immunol (2017) 18(9):1004–15. 10.1038/ni.3800 28759001

[14] Cortez VS Ulland TK Cervantes-Barragan L Bando JK Robinette ML Wang Q SMAD4 impedes the conversion of NK cells into ILC1-like cells by curtailing non-canonical TGF-beta signaling. Nat Immunol (2017) 18(9):995–1003. 10.1038/ni.3809 28759002 PMC5712491

[15] Fionda C Stabile H Cerboni C Soriani A Gismondi A Cippitelli M Hitting More Birds with a Stone: Impact of TGF-beta on ILC Activity in Cancer. J Clin Med (2020) 9(1):143. 10.3390/jcm9010143 PMC7019362 31948072

